# Falls and physical function in older patients with Benign Paroxysmal Positional Vertigo (BPPV): findings from a placebo controlled, double blinded randomized control trial (RCT) investigating efficacy of vitamin D treatment in lowering the recurrence rate of BPPV

**DOI:** 10.1007/s40520-025-02938-4

**Published:** 2025-02-22

**Authors:** Xiaoting Huang, Kenneth Wei De Chua, Shirlene Pei Shi Moh, Heng Wai Yuen, David Yong Ming Low, Poongkulali Anaikatti, Arshad Iqbal, Barbara Helen Rosario

**Affiliations:** 1 Department of Geriatric Medicine, Changi General Hospital, 2 Simei Street 3, Singapore, 529889 Singapore; 2 Department of Ear Nose and Throat, Changi General Hospital, Singapore, Singapore; 3 Department of Dietetics, Changi General Hospital, Singapore, Singapore; 4 Department of Emergency Medicine, Changi General Hospital, Singapore, Singapore

**Keywords:** Falls, Older adults, BPPV, Fear of falling, Vitamin D

## Abstract

**Background:**

Benign Paroxysmal Positional Vertigo (BPPV) is the commonest cause of vertigo in older adults. Due to its high incidence in older adults presenting with falls, vestibular assessment is recommended in the World Guidelines for Falls Prevention. There is a paucity of evidence in well conducted randomised controlled trials (RCTs) to evaluate vitamin D for prevention of BPPV recurrence in relation to falls and function.

**Aims:**

Primary outcome: does vitamin D supplementation and dietary interventions in combination with standard care impact falls, fear of falling and function in patients with BPPV.

**Methods:**

Post hoc analyses of phase IIa single centre, placebo controlled, double blind RCT evaluating vitamin D supplementation with diet and Canalith Repositioning Procedure (CRP) [Group A] versus diet alone with CRP [Group B] in reducing BPPV recurrence rates and the consequent effects on falls and function.

**Results:**

53 participants were recruited. 14 were vitamin D replete at baseline (Group C) with remaining 39 randomised into Groups A and B. Group A had better 5x sit to stand time and 0.75 fewer clinical BPPV recurrences per one person year (*P* = 0.035) compared to Group B. 25% of participants who fell reported fear of falling compared to 43% in those without falls in the 12 month follow up.

**Discussion:**

Vitamin D supplementation alongside standard BPPV improved 5xchair stand test and reduced BPPV recurrence. Participants without an incident fall during follow up experience fear of falling, prompting further consideration.

**Conclusion:**

Vitamin D replacement was associated with fewer BPPV recurrences and improved function assessed with 5x chair stand test.

## Introduction

Benign Paroxysmal Positional Vertigo (BPPV) is the most common cause of vertigo in older adults [1]. It is caused by dislodged otoconia, which fall from the utricular macula into the semicircular canals causing them to move through the canals with the effect of gravity [2]. Treatment of BPPV is primarily with Canalith Repositioning Procedure (CRP) with more than 80% success rates [3]. However, BPPV can recur 10–20% of the time and some long-term follow-up studies report up to 50% recurrence rates [4–7].

Between 32 and 42% of persons over 70 experiences at least one fall annually and injuries are common [8]. The most frequently reported causes of falls are accidental or environmental causes, but dizziness, balance disorder, as well as difficulties in transferring are the second most common causes [9]. Due to the high incidence of BPPV in older adults presenting with falls, vestibular assessment, and diagnosis of BPPV and other vestibular disorders has become a recommendation in the World Guidelines for Falls Prevention and Management in Older Adults 2022 [10]. Gassmann et al. [11] found the majority (64.5%) of older participants with dizziness described a feeling of losing their balance, which was associated with gait disturbance and risk of falls. They also found daily dizziness to be associated with difficulties while walking 500 m or getting up from bed, and a longer duration of dizziness was associated with the use of walking aids.

Although the aetiology of BPPV is idiopathic in most cases, old age, female sex, cerebrovascular diseases, anxiety, bed rest, migraine, diabetes, hyperlipidaemia, hypertension, autoimmune thyroiditis, osteoporosis and vitamin D deficiency have all been suggested as potential risk factors for BPPV occurrence and BPPV is often co-morbid with other vestibular disorders as well [12–14]. Of note, the association with osteoporosis has raised much interest [15–17] and several studies have suggested that abnormal calcium and vitamin D metabolism may underlie BPPV [18–20]. Individuals with idiopathic BPPV have a higher prevalence of vitamin D deficiency [21, 22], and lower vitamin D levels are associated with higher recurrence rates [23]. A conceptual model has been proposed for the association between BPPV, osteoporosis and vitamin D^1^ and in line with these concepts, there have been trials [24, 25] and a meta-analysis [26] that supports the therapeutic effectiveness of the correction of hypovitaminosis D in patients with recurrent BPPV.

Whilst there has been robust evidence in manoeuvres for managing BPPV as well as the detrimental effects it has on older adults, there has been a paucity of evidence in well conducted randomised controlled trials to evaluate therapies for prevention of BPPV recurrence and its relation to falls and function. Till date, there has been only 1 randomised controlled trial undertaken in Asian patients to evaluate the association of vitamin D deficiency with recurrent BPPV, and the effect of vitamin D supplementation on recurrence rates [27]. Whilst it evaluated the impact on rate of falls, it did not evaluate the effect on patient’s function and did not specifically target older adults.

Based on existing evidence, we postulate that Vitamin D replacement in those Vitamin D deficiency, combined with dietary interventions, alongside standard Canalith Repositioning Procedures (CRP) will reduce exacerbations of BPPV and thereby result in functional improvement. We conducted a single centre, randomized controlled trial to assess the effectiveness of vitamin D supplementation with diet, or diet alone combined with CRP (standard clinical care) to ascertain the patient’s functional ability, postural stability, frailty status and reduction in falls and effectiveness at reducing the recurrence of BPPV.

## Methods

### Recruitment, inclusion, and exclusion criteria

**Fig. 1:**
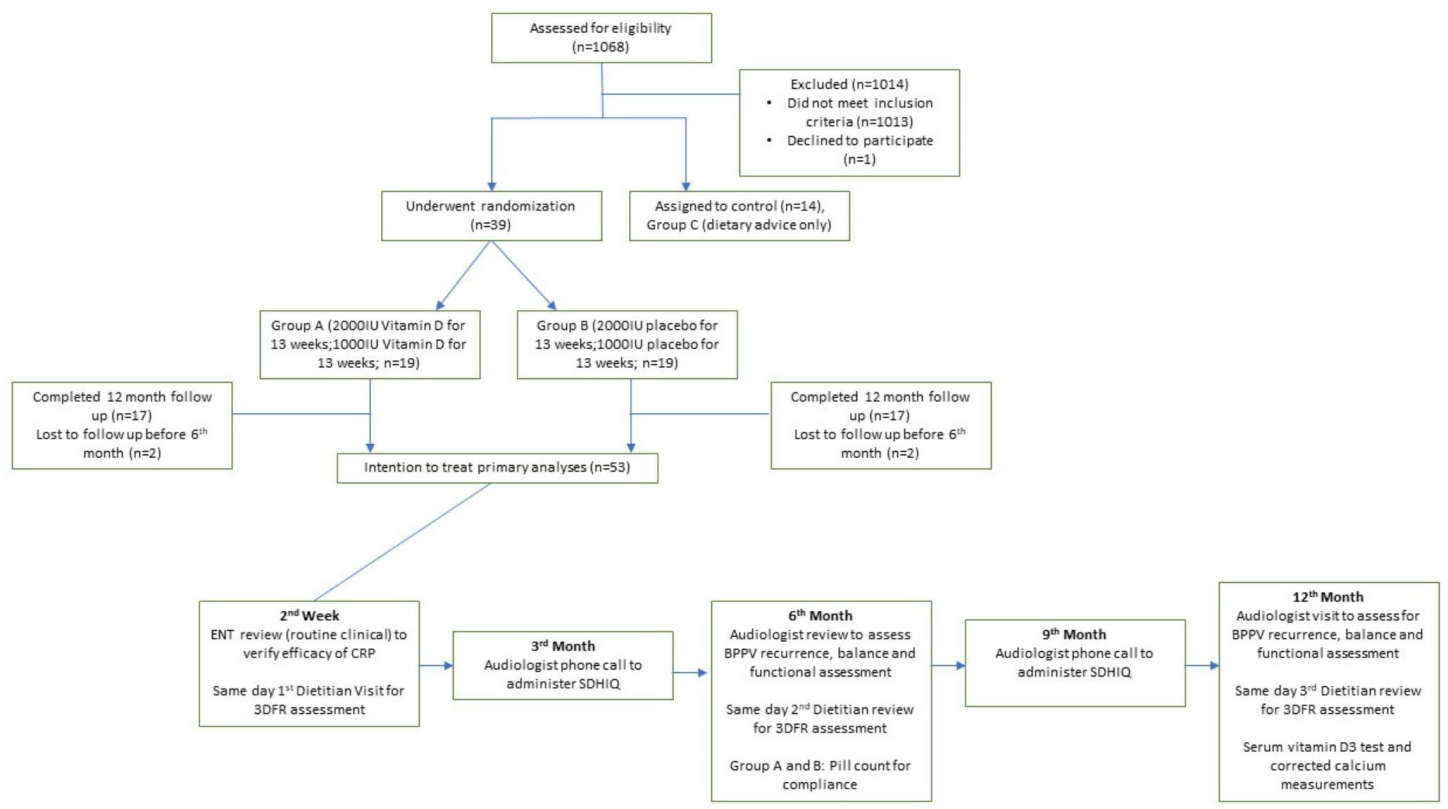
CONSORT diagram

Patient with history of giddiness suggestive of idiopathic BPPV and a positive Dix Hallpike test, were recruited either at the ENT clinic or in the Emergency Department (ED) after confirmation of the diagnosis of idiopathic BPPV by an Ear Nose and Throat (ENT) clinician and CRP as outlined in Fig. 1. Inclusion criteria were patients aged ≥ 50 years of age with a clinical history suggestive of idiopathic BPPV, supported by positive Dix Hallpike test and AMT ≥ 7. Exclusion criteria were patients with identified neurological causes of giddiness, including major neurocognitive impairment, sarcoidosis, metastatic disease (lymphoma, multiple myeloma), parathyroid disorders, known osteoporosis or osteopenia on high dose treatment (50,000 IU/week), patients with significant cervical-spinal radiculopathy, spondylolisthesis, lordosis or kyphosis that would affect their ability to carry out CRP, disorders causing fat malabsorption (Short gut syndrome, Coeliac disease) that would affect dietary absorption of Vitamin D, myasthenia gravis, patients with unexplained hypercalcaemia and pregnant women.

### Ethics and patient consent

Ethics approval was overseen by the Centralised Institutional Review Board (ethics review number 2020/2654) and regulatory approval from Health Sciences Authority (certificate number CTC2100006) prior to enrolment of patients into the study. The trial was registered under Medicines on 2020-10-09 (Clinical Trials -Trial Registration Number NCT04578470). All enrolled patients provided written informed consent for their participation and all methods included in this study are in accordance with ethical principles that have their origin in the Declaration of Helsinki.

### Objectives and trial design

This was a post-hoc analysis of data that were prospectively collected in a phase IIa single centre, placebo controlled, double blind randomized controlled trial of replacement vitamin D on benign paroxysmal positional vertigo recurrence. The primary objectives reported in this paper is to determine if vitamin D status was associated with improvements in SPPB total score, gait speed, and the 5-times sit-to-stand (5xSTS) test time, at 6 and 12 months from baseline.

Clinical teams and patients were blinded to the treatments received.

The patients were randomised into three groups depending on the result of their baseline vitamin D level. If the vitamin D level was normal, they would be allocated to group C. If vitamin D level was depleted then they would be randomised to either Group A or Group B. The patient was not informed of the treatment regime. Patients with replete Vitamin D levels (≥ 30ng/ml) serve as a control arm. All clinical investigators were blinded to the group assignments.

Group A received Vitamin D supplementation in the form of daily 2000 IU cholecalciferol (two tablets) for 13 weeks, and daily 1000 IU cholecalciferol (1 tablet) for another 13 weeks and then treatment was discontinued but dietary interventions were continued. Group B were treated with a placebo of Vitamin D with two tablets daily for 13 weeks and then 1 tablet daily for 13 weeks and dietary interventions were continued. Group C did not receive any Vitamin D interventions for the entire 12- month study period but received dietary interventions.

A total of 53 participants aged ≥ 50 years with history suggestive of idiopathic BPPV were included in this study. 39 participants with < 30ng/ml vitamin D at baseline were randomly assigned in a 1:1 allocation ratio to group A (*n* = 19) and B (*n* = 20).

### Patient assessments and follow up

Baseline assessments included a serum vitamin D3 test and corrected calcium measurements and abbreviated mental test (AMT). Balance and functional assessment in the form of a Gans Sensory Organisation Performance Test (Gans SOP), Short Physical Performance Battery (SPPB), Barthel Index (BI) and Rockwood Clinical Frailty Scale (CFS) were undertaken. Falls history in the prior 12months was taken including any fear of falling. Follow ups for all 3 groups included a physical clinic review at 1–2 weeks, 3 month tele-consult, 6 month physical review by audiologist and dietitian, 9 month tele-consult and 12 month physical review by audiologist and dietitian.

Compliance to medications was checked during the 6-month physical review where randomised patients were requested to bring in their prescribed medications for a final compliance pill count. BPPV recurrence was assessed during tele-consults using questions from the Shortened Dizziness Handicap Inventory Questionnaire (SDHIQ). At physical reviews at the 6- and 12-month mark, patients were interviewed on symptoms relating to falls and fear of falling and BPPV recurrence and underwent Dix Hallpike test, balance and functional assessment in the form of GANS SOP, SPPB, and BI. All groups underwent a final serum vitamin D3 test and corrected calcium measurements at the 12-month visit.

### Statistical analyses

The reporting of study results was in accordance with STROBE guidelines [28]. Descriptive statistics of patient demographic and clinical characteristics were reported as number and percent for categorical data, mean ± standard deviation (SD) for normally distributed data, and median and interquartile range (IQR) for non-normally distributed data.

Baseline was defined as the date of randomization for participants who were randomized to group A or B, and the date of first Dix-Hallpike measurement for patients who were non-randomized in group C. Subgroup analyses by frailty status and fear of falling at baseline were also performed. Statistical tests were two-sided with a 0.05 significance level. All statistical analyses were conducted using Stata 18 (College Station, TX: StataCorp LLC).

## Results

A total of 53 participants aged ≥ 50 years with history suggestive of idiopathic BPPV were included in this study. There were 14 non-randomized participants with ≥ 30ng/ml baseline vitamin D who were assigned to group C and 39 participants randomized in a 1:1 ratio to group A (*n* = 19) and B (*n* = 20). The mean age of all 53 participants was 66.2 ± 8.1 years and 66.0% (35/53) of participants were female. Table 1 shows the baseline demographic and clinical characteristics overall, and by group. The predominance of females in this study is consistent with previous literature supporting a higher prevalence of BPPV in female older adults [27]. The study population was relatively robust with 31 (59.6%) of the participants at CFS 3 (Managing Well) with a gait speed of > 0.8 m/s.

**Table 1:** Baseline demographic and clinical characteristics of participants

Characteristic	All participants (*n* = 53)	Group A (*n* = 19) Treatment grp	Group B (*n* = 20) Placebo grp	Group C Non-randomized participants [vitamin D replete] (*n* = 14)
Age in years, mean ± SD	66.2 ± 8.1	66.5 ± 9.4	66.5 ± 8.1	65.6 ± 6.4
Age group in years, n (%)				
50–59	12 (22.6)	5 (26.3)	4 (20)	3 (21.4)
60–69	23 (43.4)	6 (31.6)	10 (50)	7 (50)
70–79	16 (30.2)	7 (36.8)	5 (25)	4 (28.6)
≥80	2 (3.8)	1 (5.3)	1 (5)	0 (0)
Female gender, n (%)	35 (66.0)	15 (79.0)	12 (60)	8 (57.1)
BMI in kg/m^2^	22.4 ± 9.6	21.3 ± 11.3	24.0 ± 9.6	21.7 ± 7.4
Fear of falling, n (%)	22 (47.8)	3 (21.4)	10 (55.6)	9 (64.3)
Fell in the past year, n (%)	2 (4.4)	0 (0)	2 (11.1)	0 (0)
History of fall, n (%)	2 (3.8)	0 (0)	2 (10)	0 (0)
Dix–Hallpike test result, n (%)				
Positive	31 (60.8)	8 (44.4)	15 (75)	8 (61.5)
Negative	20 (39.2)	10 (55.6)	5 (25)	5 (38.5)
Barthel Index for ADL score				
Mean ± SD	19.7 ± 0.8	19.9 ± 0.3	19.5 ± 1	19.8 ± 0.8
Median (IQR)	20 (20–20)	20 (20–20)	20 (19.5–20)	20 (20–20)
SPPB				
Side-by-side stand, n (%)				
Held for 10s	53 (100)	19 (100)	20 (100)	14 (100)
Not held for 10s	0 (0)	0 (0)	0 (0)	0 (0)
Semi-tandem stand, n (%)				
Held for 10s	49 (92.5)	19 (100)	17 (85)	13 (92.9)
Not held for 10s	1 (1.9)	0 (0)	1 (5)	0 (0)
Not attempted	3 (5.7)	0 (0)	2 (10)	1 (7.1)
Tandem stand, n (%)				
Held for 10s	36 (67.9)	14 (73.7)	13 (65)	9 (64.3)
Held for 3 to 9.99s	7 (13.2)	1 (5.3)	3 (15)	3 (21.4)
Held for < 3s	5 (9.4)	2 (10.5)	2 (10)	1 (7.1)
Not attempted	5 (9.4)	2 (10.5)	2 (10)	1 (7.1)
Balance test total score, n (%)				
4	36 (67.9)	14 (73.7)	13 (65)	9 (64.3)
3	7 (13.2)	1 (5.3)	3 (15)	3 (21.4)
2	6 (11.3)	4 (21.1)	1 (5)	1 (7.1)
1	4 (7.6)	0 (0)	3 (15)	1 (7.1)
Gait speed test				
Time in s, n (%)				
Median (IQR)	2.7 (2.4–3.4)	2.9 (2.3–3.4)	2.9 (2.6–3.4)	2.6 (2.2–3.6)
<3.62	39 (78)	15 (83.3)	14 (77.8)	10 (71.4)
3.62–4.65	9 (18)	3 (16.7)	3 (16.7)	3 (21.4)
4.66–6.52	1 (2)	0 (0)	0 (0)	1 (7.1)
>6.52	1 (2)	0 (0)	1 (5.6)	0 (0)
5xSTS time in secs, median (IQR)	9.4 (8.2–11.7)	8.9 (8.1–10.9)	9.6 (9.1–12.6)	9.0 (7.5–11.9)
Chair stand test score, median (IQR)	4 (3–4)	4 (4–4)	4 (3–4)	4 (3–4)
SPPB total score, median (IQR)	11 (10–12)	12 (10–12)	11 (9–12)	11 (10–12)
DHI-S, median (IQR)				
Physical sub-score	6 (4–9)	4 (2–10)	6 (4–8)	8 (6–10)
Functional sub-score	2 (0–4)	2 (0–4)	2 (0–4)	2 (2–2)
Total score	8 (5–12)	6 (2–14)	8 (6–10)	10 (8–12)
CFS score, n (%)				
1	2 (3.9)	0 (0)	0 (0)	2 (14.3)
2	14 (26.9)	7 (38.9)	6 (30)	1 (7.1)
3	31 (59.6)	10 (55.6)	11 (55)	10 (71.4)
4	5 (9.6)	1 (5.6)	3 (15)	1 (7.1)
5–9	0 (0)	0 (0)	0 (0)	0 (0)
Medical history, n (%)				
Myocardial infarction	1 (1.9)	0 (0)	1 (5)	0 (0)
CHF	0 (0)	0 (0)	0 (0)	0 (0)
Peripheral vascular disease	0 (0)	0 (0)	0 (0)	0 (0)
TIA or CVA	1 (1.9)	0 (0)	1 (5)	0 (0)
Chronic cognitive deficit	0 (0)	0 (0)	0 (0)	0 (0)
COPD	0 (0)	0 (0)	0 (0)	0 (0)
Connective tissue disease	0 (0)	0 (0)	0 (0)	0 (0)
Peptic ulcer disease	0 (0)	0 (0)	0 (0)	0 (0)
Hemiplegia	0 (0)	0 (0)	0 (0)	0 (0)
Solid tumor	1 (1.9)	0 (0)	1 (5)	0 (0)
Leukemia	0 (0)	0 (0)	0 (0)	0 (0)
Lymphoma	0 (0)	0 (0)	0 (0)	0 (0)
AIDS	0 (0)	0 (0)	0 (0)	0 (0)
Moderate to severe CKD	2 (3.8)	1 (5.3)	1 (5)	0 (0)
Liver disease	0 (0)	0 (0)	0 (0)	0 (0)
Diabetes mellitus	17 (32.1)	8 (42.1)	4 (20)	5 (35.7)
Biochemical marker, mean ± SD				
Calcium in mmol/L	2.23 ± 0.10	2.25 ± 0.12	2.20 ± 0.08	2.23 ± 0.08
Albumin in g/L	46.0 ± 3.1	46.1 ± 2.5	44.6 ± 3.4	47.9 ± 2.5
25-hydroxyvitamin D_3_ in ng/mL	24.1 ± 8.8	20.7 ± 5.5	19.5 ± 7.1	35.2 ± 3.5

5xSTS = 5-times sit-to-stand. ADL = Activities of daily living. AIDS = Acquired immunodeficiency syndrome. BMI = Body mass index. CFS = Clinical frailty scale. CHF = Congestive heart failure. CKD = Chronic kidney disease. COPD = Chronic obstructive pulmonary disease. CVA = Cerebrovascular accident. DHI-S = Dizziness handicap inventory, short version. GRM = Gans repositioning maneuver. IQR = Interquartile range. MRT = Mass rapid transit (Singapore). SD = Standard deviation. SPPB = Short physical performance battery. TIA = Transient ischemic attack

10.5% (2/19) of participants in group A were lost to follow-up before 6 months post randomization, while 5% (1/20) in group B were lost to follow-up before 12 months post randomization. Participants were analysed based on original group assignment with intention to treat protocol and vitamin D replacement in Group A was associated with 0.75 fewer clinical BPPV recurrences per one person year [29]. Based on our previous findings, Group C also had higher recurrences of BPPV compared to Group A although it did not reach clinical significance. This is not surprising given that baseline vitamin D status in Group C is also replete. Most participants did not have a history of prior falls in the year prior to the study. Of the participants who experienced falls during the 12 month follow up (*n* = 4), only 25% (*n* = 1) reported fear of falling at baseline. Interestingly, out of participants with no incident fall during the 12 month follow up (*n* = 49), 43% had a fear of falling (Table 2).

**Table 2:** Factors associated with incident fall

Factor	Participants with no incident fall during 12-month follow-up (*n* = 49)	Participants with incident fall during 12-month follow-up (*n* = 4)	*P* value*
Original group assignment, n (%)			0.664
A	18 (33.3)	1 (25)	
B	18 (33.3)	2 (50)	
C	13 (24.0)	1 (25)	
Demographic			
Age at baseline, mean ± SD	66.1 ± 8.1	68 ± 9.1	0.713
Female gender, n (%)	32 (65.3)	3 (75)	1.000
Fall-related, n (%)			
Fell in the past year	2 (4.8)	0 (0)	1.000
Fear of falling at baseline	21 (43)	1 (25)	0.609
Measure of functional mobility at baseline, median (IQR)			
Barthel Index for ADL score	20 (20–20)	19.5 (18–20)	0.043
SPPB total score	11 (10–12)	10 (8–10.5)	0.067
25-hydroxyvitamin D_3_ at baseline in ng/mL, mean ± SD	23.9 ± 8.7	26.1 ± 11.7	0.734

*P values were based on Fisher’s exact test for categorical factors, Welch’s t-test for continuous factors that were approximately normally distributed, and the Mann-Whitney U-test for factors that were not normally distributed

IQR = Interquartile range. SD = Standard deviation. SPPB = Short physical performance battery

There was also a low incidence of falls in this study group with a total of 4 falls across Groups A-C in the 12 month follow up period (Table 2). Out of the 4 who had a fall, 1 was in group C which were participants with baseline replete vitamin D levels, 2 were in group B, 1 was in group A.

Across participants in group A, compliance rates ranged from 64.7 to 100% with 3 drop-outs. At month 12, despite being on vitamin D replacement, the participant in Group A who fell had a vitamin D level of 25.4 from a baseline of 20.7 despite a 100% compliance rate.

For the 2 participants in Group B who fell, one of them had a vitamin D level of only 12.3 at the start of study and no end point vitamin D levels as the patient was lost to follow up. The other participant in group B started with a vitamin D level of 11.1 and had a level of 12 at the end of the study. As for the participant in group C, vitamin D levels went from 34.5 to 29.9 with dietary interventions alone.

Compared to participants with no incident fall, participants with an incident fall have a lower baseline Barthel Index for ADL score (*P* = 0.043; Table 2) and baseline SPPB total score (median 10 vs. 11, *P* = 0.067; Table 2).

**Table 3:** Original group assignment on SPPB score at 6 and 12 months (Group A and B). Original group assignment on SPPB score at 6 and 12 months

Subgroup and outcome	Group A	Group B	Group C	Group A vs. Group B, *P* value*	Group A vs. Group C, *P* value*
**All participants (A: 19, B: 20, C: 14)**					
SPPB at 6 months					
Total score	12 (12–12)	12 (10–12)	12 (12–12)	0.287	0.632
Gait speed test time in s	2.77 (2.39–3.55)	3 (2.47–3.35)	2.82 (2.52–3.20)	0.618	0.824
5xSTS time in mins	8.98 (7.52–10.42)	9.09 (8.11 to 10.93)	8.10 (7.99–9.46)	0.356	0.929
SPPB at 12 months					
Total score	12 (12–12)	12 (10–12)	12 (11.5–12)	0.264	1.000
Gait speed test time in s	2.89 (2.55 to 3.29)	2.65 (2.53 to 3.23)	2.72 (2.43–2.91)	0.967	0.413
5xSTS time in mins	8.52 (7.86 to 10.51)	10.25 (7.92 to 12.3)	9.03 (7.74–10.41)	0.112	0.664
**Participants with baseline CFS score of 2 (A: 7**,** B: 6)**					
SPPB at 6 months					
Total score	12 (11–12)	12 (12–12)	–	0.845	–
Gait speed test time in s	2.36 (1.95–4.11)	2.87 (2.45–3.32)	–	0.655	–
5xSTS time in mins	8.77 (7.11 to 9.18)	8.59 (8.37 to 9.72)	–	0.655	–
SPPB at 12 months					
Total score	12 (12–12)	12 (11.5–12)	–	0.317	–
Gait speed test time in s	2.31 (2.17–2.65)	2.64 (2.41 to 3.00)	–	0.149	–
5xSTS time in mins	8.27 (6.42 to 9.53)	9.48 (7.92 to 10.37)	–	0.724	–
**Participants with baseline CFS score of 3 or 4 (A: 12**,** B: 14)**					
SPPB at 6 months					
Total score	12 (12–12)	12 (8–12)	–	0.177	–
Gait speed test time in s	2.36 (1.95–4.11)	2.87 (2.45–3.32)	–	0.626	–
5xSTS time in mins	9.25 (7.52–10.49)	9.59 (7.87–11.37)	–	0.468	–
SPPB at 12 months					
Total score	12 (11–12)	11.5 (10–12)	–	0.437	–
Gait speed test time in s	3.01 (2.78–3.33)	2.65 (2.59–3.37)	–	0.468	–
5xSTS time in mins	8.94 (7.86–10.51)	11.15 (8.40–13.13)	–	0.136	–
**Participants who reported no fear of falling (A: 11**,** B: 8)**					
SPPB at 6 months					
Total score	12 (11.5–12)	12 (11–12)	–	0.533	–
Gait speed test time in s	2.83 (2.38–3.56)	2.87 (2.45–3.35)	–	1.000	–
5xSTS time in mins	8.98 (7.32–10.46)	9.59 (8.05–11.37)	–	0.643	–
SPPB at 12 months					
Total score	12 (12–12)	12 (11–12)	–	0.189	–
Gait speed test time in s	2.89 (2.32–3.12)	2.65 (2.47–2.93)	–	0.770	–
5xSTS time in mins	8.71 (7.86–10.51)	10.20 (9.48–12.06)	–	0.083	–

All data were reported as median (IQR). SPPB scores were excluded for subgroups of participants in group C due to the small sample size

*P values of Mann-Whitney U-test

5xSTS = 5-times sit-to-stand. CFS = Clinical Frailty Scale. SPPB = Short physical performance battery

There was no evidence of a difference in SPPB scores at 6 or 12 months across all groups (Table 3).

Participants who received vitamin D supplementation in group A had a better 5x sit to stand time compared to those in Group B as seen in Table 3. This finding was consistent across all subgroups in participants with CFS score of 2, participants with CFS score of 3 or 4 and participants with fear of falling. SPBB scores remained similar across Groups A and B with a small decline in participants with baseline CFS score of 4 and 3 from 12 to 11.5 from 6 to 12 months, although it did not reach statistical significance.

## Discussion

Falls have been shown in studies to be an indicator of complex system failure in our older adults rather than a single isolated organ dysfunction, congruent with existing guidelines for multifactorial contributions [10]. The significant reduction in clinical BPPV recurrence rate in group A (vitamin D) compared to group B (placebo) suggests a potential protective effect of vitamin D supplementation against BPPV recurrences in our earlier study [29] however, interestingly, this this not translate to fewer falls in post hoc analysis as evident in Table 2, highlighting that there may be further contributing factors to falls in this cohort that is relatively robust with few falls at baseline. A systematic review in 2023 published by Pauwels et al. [30] noted that BPPV increases the odds of falling and negatively impacts spatiotemporal parameters of gait as a potential mediating factor for falls. Another study by Hawke et al. [31] also notes the high frequency of BPPV that are undiagnosed in patients with high falls risk and an increased loneliness in this group of patients, likely due to self imposed physical limitations due to BPPV.

The study population only had 2 participants who had a history of falls. Participants with no incident fall during the 12 month follow up period, had a higher fear of falling at baseline (*n* = 21) compared to participants who fell (*n* = 1) (Table 2). However, there is a low prevalence of falls in this study population and the results did not reach significance. Furthermore, as mentioned at baseline, the study population was relatively robust with majority having a CFS score of 3 and gait speed of > 0.8 m/s and only five (9.6%) very mildly frail individuals.

It has been shown in literature that about 30–50% of older adults who live independently experience fear of falling regardless of whether they had prior falls [32, 33]. Whilst previous studies have shown that fear of falling can lead to increased risk of future falls, this was not apparent in our study population [32, 33, 34]. Fear of falling has also been associated with reduction in functional status through maladaptive mechanisms such as activity restriction and decreased independence [37–37] which we also see in our study population where the Barthel index was 20 compared to 19.5 (Table 3) although it did not reach statistical significance since our study population were functionally robust.

A recent study published by Lee et al. [38] undertook a concept analysis into fear of falling and noted that it can lead to five main consequences: protective effect, activities curtailment, reduction in radius of living, restricted freedom, and limited social activities. The idea of fear of falling as being protective has been also explored in other studies [39, 40] as it helps older adults attract attention to potential risks, which can increase concentration on the task and maintain the correct movements required to maintain safety– which may explain why participants with no incident fall during the 12 month follow up had higher fear of falling at baseline. The theory of fear of falling is complex and warrants further quantitative and qualitative evaluation to better characterize the consequences and interventions to reduce risk of future falls.

As mentioned, compliance rates in Group A ranged from 64.7 to 100% however, there appears to be poorer than expected response to vitamin D replacement in this study population. Emerging research has shown that factors such as ambient UV-B, age, sex, body mass index, cholesterol level and ethnicity can influence response to vitamin D supplementation [41, 42] which is relevant in Singapore as a multiethnic society.

In addition, the finding of improved 5x sit to stand time across groups in Table 3 supports existing evidence for vitamin D replacement for patients who are deficient in muscle function [43]. This brings up further question with regards to the group who will benefit the greatest from vitamin D supplementation. An update on Vitamin D for the Prevention of Disease by the Endocrine Society [44] in 2024 recommend against vitamin D supplementation for older adults aged 50–74 years old. However, it was also mentioned that the reason for this recommendation was that recent clinical trials did not support findings of benefit of vitamin D supplementation [45, 38], perhaps because many participants in these trials did not have low baseline 25(OH)D levels. This is also supported in our current findings where participants with low vitamin D levels in group A had improved physical performance after receiving vitamin D supplementation, even if not all reached level of sufficiency (i.e. > 30ng/dL) at the end of the 12 month follow up.

Our study does have its own limitations. This is a single center placebo controlled, double blinded randomized control trial (RCT) investigating efficacy of Vitamin D treatment in lowering the recurrence rate of BPPV which evaluated the relationship between falls and function. The study cohort is also small with only 53 participants. As this study was conducted during the COVID period, hybrid follow up of physical reviews and tele-consults were used which may have affected accuracy of reporting of falls and near falls. As mentioned, the study population was relatively robust, limiting the generalizability of the study to our frailer community dwelling adults.

It would also have been good to evaluate the effect of diet alone on vitamin D status in future studies. In our study, although participants in group A were treated with a total of 26 weeks of vitamin D replacement, we do not have vitamin D levels mid study to evaluate the effect of dietary interventions alone on maintaining vitamin D levels.

## Conclusion

Our single centre randomised control trial in a robust population of community dwelling older adults found that vitamin D supplementation improved physical performance in 5x chair stand test. Furthermore, even with a small study cohort, we have shown that vitamin D replacement was associated with 0.75 fewer clinical BPPV recurrences per one person year. There was overall low falls prevalence at baseline in the study population and incident falls on 12 month follow up. In this study population, more participants without an incident fall during follow up experienced fear of falling, prompting further consideration into the complex concept that is fear of falling. This paper has highlighted a few questions for further research such as the factors affecting vitamin D absorption from diet and potential positive effect of fear of falling in preventing falls in robust individuals.

## Data Availability

No datasets were generated or analysed during the current study.
